# Senotherapeutics for Brain Aging Management

**DOI:** 10.3390/neurolint17120204

**Published:** 2025-12-15

**Authors:** Timur Saliev, Prim B. Singh

**Affiliations:** 1 School of Medicine, Nazarbayev University, Astana 010000, Kazakhstan; 2 Institute of Fundamental and Applied Medical Research, S.D. Asfendiyarov Kazakh National Medical University, Almaty 050000, Kazakhstan

**Keywords:** brain aging, inflammation, neurodegenerative disease, pro-inflammatory secretory profile (SASP), senotherapeutics, senolytics, senomorphics

## Abstract

Brain aging is a progressive process marked by cellular dysfunction, chronic inflammation, and increased susceptibility to neurodegenerative diseases. A growing body of evidence identifies cellular senescence, the accumulation of non-dividing, metabolically active cells with a pro-inflammatory secretory profile (SASP), as a key contributor to cognitive decline and brain aging. This review explores the emerging field of senotherapeutics, which includes senolytics (agents that eliminate senescent cells) and senomorphics (agents that suppress SASP without killing cells), as potential strategies to manage brain aging. We summarize recent preclinical studies demonstrating that senotherapeutics can reduce neuro-inflammation, improve synaptic plasticity, and enhance cognitive function in aged animal models. Additionally, we highlight early-phase clinical trials investigating senolytic compounds in Alzheimer’s disease and discuss key challenges, including the delivery of drugs to the brain, biomarker development, and long-term safety. The review concludes that senotherapeutics, particularly when combined with personalized and multimodal approaches, represent a promising avenue for mitigating age-related cognitive decline and promoting healthy brain aging.

## 1. Introduction

The global increase in life expectancy has led to a dramatic rise in age-associated cognitive decline and neurodegenerative disorders, such as Alzheimer’s disease (AD), Parkinson’s disease (PD), and vascular dementia [1,2]. These conditions not only affect the quality of life for aging individuals but also place an immense burden on healthcare systems. Despite this growing challenge, effective strategies to prevent or delay brain aging and its associated pathologies remain limited. Traditional approaches have largely focused on managing symptoms rather than addressing the underlying biological drivers of brain aging [3]. Recent advances in the biology of aging, however, have revealed new therapeutic targets, with cellular senescence emerging as a central mechanism of neural deterioration [4,5].

Cellular senescence is a state of stable cell cycle arrest, initially identified in proliferative cells, accompanied by persistent metabolic activity and the secretion of a pro-inflammatory cocktail of molecules known as the senescence-associated secretory phenotype (SASP) [6,7]. The dual nature of cellular senescence is now a key therapeutic target in aging research. Initially, it acts as a beneficial mechanism by halting the proliferation of damaged cells, thus suppressing tumor development, and facilitating wound repair through the coordinated release of specific factors.

The pathology arises from the chronic accumulation of these senescent cells. Their persistent pro-inflammatory secretion (SASP), now often called the Senescence Messaging Secretome), creates a toxic tissue environment. In the brain, the accumulation of senescent microglia and astrocytes is a major driver of neuroinflammation. Recent studies directly link this process to cognitive decline and neurodegenerative pathologies, making the clearance of senescent cells (senolysis) a promising strategy to combat brain aging [8]. Ogrodnik et al., (2021) provided foundational evidence that systemic senolytic clearance can directly alleviate brain inflammation and cognitive decline in aged mice [9]. Most recently, Suda et al., (2024) introduced a groundbreaking prophylactic approach, a senolytic vaccine, that successfully reduced age-related pathologies, including metabolic dysfunction, and extended lifespan in progeroid mice, highlighting the immense therapeutic potential of targeting senescence [10].

In the central nervous system (CNS), senescent cells, including astrocytes, microglia, oligodendrocyte progenitors, and endothelial cells, accumulate with age and secrete SASP factors [11]. It disrupts neuronal homeostasis, impair synaptic function, and weaken the blood–brain barrier [12]. These changes contribute to reduced cognitive resilience, increased susceptibility to neurodegeneration, and progression of diseases like AD and PD.

The identification of senescence as a modifiable factor in brain aging has led to the emergence of senotherapeutics, a new class of pharmacological interventions aimed at either eliminating senescent cells (senolytics) or modulating their harmful secretory profile (senomorphics) [13]. Senolytics, such as dasatinib and quercetin, selectively induce apoptosis in senescent cells by targeting pro-survival pathways unique to the senescent state [14].

Dasatinib is an FDA-approved oral tyrosine kinase inhibitor used in the treatment of Philadelphia chromosome-positive leukemias, including chronic myeloid leukemia (CML) in all disease phases and Philadelphia chromosome–positive acute lymphoblastic leukaemia (Ph+ ALL) in patients resistant or intolerant to prior therapy [15]. Its mechanism of action is based on potent inhibition of multiple kinases, primarily BCR-ABL, the fusion protein driving leukemic proliferation, as well as SRC family kinases, c-KIT, and PDGFR, which contribute to tumor cell survival and progression. By blocking these signaling pathways, dasatinib induces apoptosis and inhibits cell growth in malignant hematopoietic cells [16]. Beyond its established oncological use, dasatinib has demonstrated broader biological activity, including immunomodulatory, anti-inflammatory, and anti-fibrotic effects. Rodríguez-Agustín and colleagues are have highlighted dasatinib as an agent capable of modulating the immune system by expanding memory-like NK cells and γδ T cells, contributing to enhanced viral control and improved outcomes in CML and HIV [16]. The authors also emphasize dasatinib’s growing recognition as a senolytic drug capable of clearing senescent cells and attenuating the chronic inflammatory milieu associated with aging and age-related diseases. This broad view positions dasatinib as a molecule with significant potential in immunotherapy, viral eradication strategies, and geroscience.

In addition, the experimental work of Wichaiyo et al. demonstrates a regenerative effect of dasatinib when applied topically in a mouse wound-healing model [17]. Their findings show that dasatinib ointment accelerates wound closure, reduces inflammatory cell infiltration, enhances angiogenesis, and promotes keratinocyte proliferation. These effects underscore that dasatinib’s biological actions are strongly influenced by dosage and route of administration, and that locally delivered dasatinib can produce pro-healing effects distinct from its systemic activity. This work expands the therapeutic horizon of dasatinib into dermatology and tissue repair, suggesting a potential for repurposing in regenerative medicine.

The study by Lachota et al. focuses on dasatinib’s influence on NK cell function and tumor immunity [18]. Their results reveal a complex, time-dependent relationship: while short-term dasatinib exposure suppresses NK cell degranulation and cytokine production, prolonged pre-treatment can enhance NK cytotoxicity ex vivo. However, in mouse models, high systemic doses of dasatinib result in increased tumor growth, likely due to suppressed NK cell activity in vivo. These findings highlight the nuanced and sometimes contradictory immunological effects of dasatinib, reinforcing the importance of exposure timing and dosing strategy when considering dasatinib in immune-sensitive clinical settings.

Finally, Chang et al. provide mechanistic evidence linking dasatinib’s immunomodulatory actions to improved clinical outcomes [19]. They demonstrate that dasatinib selectively down-regulates the inhibitory receptor NKG2A on CD56^dim NK cells, thereby enhancing NK-mediated cytotoxicity. Their clinical observations show that CML patients treated with dasatinib who exhibit lower NKG2A expression achieve major molecular responses more rapidly than those on other TKIs. The authors identify involvement of the p38 MAPK pathway and altered GATA-3 activity, offering a mechanistic explanation for dasatinib’s superior immunological and clinical performance.

Quercetin is a naturally occurring flavonoid widely found in fruits, vegetables, and plants such as onions, apples, and berries [20]. Although not approved by the FDA as a pharmaceutical drug, it is recognized as a safe dietary supplement with potent antioxidant, anti-inflammatory, and cytoprotective properties [21,22,23]. Quercetin exerts its biological effects by modulating key signaling pathways, including NF-κB, MAPK, and PI3K/AKT, thereby reducing oxidative stress, inhibiting pro-inflammatory cytokine production, and promoting cellular homeostasis [24,25,26,27]. It also influences mitochondrial function and supports vascular health, contributing to improved metabolic and cardiovascular outcomes [28]. In experimental and translational studies, quercetin has demonstrated anticancer, antiviral, and neuroprotective activities, reflecting its broad pharmacological potential [29,30].

Within the field of aging research, quercetin is recognized as both a senomorphic and senolytic compound, capable of suppressing the pro-inflammatory secretory phenotype of senescent cells and, when used in combination with dasatinib, selectively inducing their apoptosis. The study by Torikul Islam and colleagues (2023) explores the senolytic combination dasatinib plus quercetin (D & Q) in aged mice, demonstrating potent anti-senescent and anti-inflammatory effects primarily in perigonadal white adipose tissue [14]. Their findings show significant reductions in senescence markers (p16, p21, SA-β-gal) and SASP cytokines (including IL-6, TNF-α, MCP-1), accompanied by decreased immune cell infiltration. Importantly, D & Q treatment improved fasting glucose, glucose tolerance, hepatic gluconeogenesis markers, and systemic lipid metabolism. A strength of the study lies in demonstrating that senolytic-mediated reduction in adipose inflammation translates into systemic metabolic improvements, suggesting a mechanistic link between senescence, chronic inflammation, and age-related metabolic dysfunction.

In contrast, Sun et al., (2024) focus on bone marrow mesenchymal stem cells (BMSCs), where quercetin alone exerts strong senomorphic and senolytic effects by stabilizing heterochromatin and suppressing repetitive element (RE)-triggered innate immune activation [31]. Their work identifies a previously underappreciated pathway: activation of repetitive elements leading to cytoplasmic dsRNA accumulation and subsequent triggering of the RIG-I RNA sensing pathway in senescent BMSCs. Quercetin attenuated this cascade by restoring epigenetic silencers such as H3K9 methylation, HP1α, and LAP2, thereby reducing senescence markers and restoring osteogenic potential. Notably, RIG-I knockdown mimicked quercetin’s protective effects, confirming the pathway’s functional relevance. This study highlights that quercetin’s anti-aging effects extend beyond antioxidant or anti-inflammatory actions, involving epigenetic preservation and innate immune modulation.

Finally, the study by Iswarya, B.R. and John, C.M., (2025) investigates quercetin and hesperidin in the context of cancer therapy–induced senescence (TIS) in A549 lung adenocarcinoma cells [32]. 5-Fluorouracil exposure induces senescence and a SASP that fosters chemoresistance and enhanced migration. Both quercetin and hesperidin act as senolytics in this system, reducing SASP markers, suppressing paracrine resistance, and selectively promoting apoptosis in senescent cells. Hesperidin exhibits stronger senolytic activity than quercetin, particularly in boosting p53 activation and reducing colony formation. In this malignant context, quercetin’s role is more nuanced: rather than simply reducing inflammation or stabilizing tissue homeostasis, it functions by selectively clearing TIS cells and reversing SASP-driven chemoresistance, positioning flavonoids as adjunct agents in oncology.

Despite differences in models, aging metabolism (Islam et al. [14]), stem cell aging (Sun et al. [31]), and cancer TIS (Iswarya & John [32]), all three studies underscore quercetin’s versatility as a senotherapeutic agent. Together, they illustrate that quercetin and related flavonoids modulate senescence through three overlapping mechanisms: (1) direct clearance of senescent cells (senolysis), (2) suppression of SASP signaling (senomorphism), and (3) correction of upstream molecular drivers (epigenetic instability, dsRNA sensing, inflammation). These complementary findings support the broader translational potential of quercetin-based therapies across metabolic disease, regenerative medicine, and oncology, while highlighting the need to tailor senotherapeutic strategies to tissue-specific mechanisms and cellular contexts.

Senomorphics, including rapamycin and metformin, suppress the SASP, reducing inflammation and preserving tissue integrity without inducing cell death [33]. Both classes of agents have shown promising effects in preclinical models of aging and neurodegeneration, improving cognitive outcomes and reducing neuroinflammation.

However, translating these findings into clinical practice presents several challenges, including drug delivery across the blood–brain barrier, identification of reliable senescence biomarkers, and understanding the long-term safety of senotherapeutic interventions in humans [34]. Furthermore, senescent cell heterogeneity in the brain and their context-dependent roles complicate therapeutic strategies. Nevertheless, advances in molecular diagnostics, drug delivery technologies, and systems biology offer new avenues to optimize senotherapeutic approaches for brain aging.

This review aims to provide a comprehensive overview of senotherapeutics in the context of brain aging, examining the role of cellular senescence in cognitive decline, evaluating current preclinical and clinical evidence for senolytics and senomorphics, and exploring the future directions and challenges in this emerging field. By integrating recent discoveries with therapeutic innovation, senotherapeutics hold potential as a transformative strategy for promoting healthy brain aging and reducing the burden of neurodegenerative diseases.

## 2. Cellular Senescence and the Aging Brain

Cellular senescence was initially characterized in proliferative somatic cells, such as fibroblasts, as an irreversible cell cycle arrest in response to damage, serving as a barrier against cancer. However, research over the past decade has fundamentally expanded this view, revealing that senescence is not confined to dividing cells [35]. Critically, post-mitotic cells (like neurons) and slowly dividing glial cells (like astrocytes and microglia) in the CNS can also enter a senescent-like state.

In these CNS cells, the hallmark is not a proliferative arrest but a profound shift in cellular function and secretome. These cells become metabolically hyperactive and adopt a persistent pro-inflammatory and tissue-destructive profile known as the SASP. This state is characterized by dysregulated lysosomal activity (evidenced by Senescence-Associated β-Galactosidase, SA-β-Gal), sustained DNA damage response, and the upregulation of cyclin-dependent kinase inhibitors like p16INK4a and p21CIP1/WAF1 [36,37,38]. The accumulation of these dysfunctional, senescent glial and neuronal cells is a key driver of the chronic neuroinflammation and tissue decline observed in brain aging.

A particularly detrimental aspect of the SASP is its ability to propagate the senescent phenotype to neighboring healthy cells, a process known as the “bystander effect” [39]. This spreading occurs through several mechanisms. SASP factors, such as pro-inflammatory cytokines (IL-6, IL-1β) and reactive oxygen species (ROS), can induce DNA damage and stress signaling in nearby cells, pushing them into senescence. Furthermore, senescent cells release extracellular vesicles (exosomes) that package and deliver pro-senescence signals, including specific miRNAs, proteins, and even damaged DNA, directly to recipient cells. This paracrine signaling creates a feed-forward, vicious cycle where initial pockets of senescent cells can amplify tissue dysfunction, leading to a more widespread loss of tissue homeostasis in the aging brain.

Recent research has significantly broadened our understanding of senescence, demonstrating that it is not restricted to dividing cells. Indeed, post-mitotic and slowly dividing cells in the CNS, including neurons, astrocytes, microglia, oligodendrocyte progenitor cells (OPCs), pericytes, and cerebral endothelial cells, can also enter a senescent-like state [40,41,42]. In these non-dividing cells, senescence does not result in cell cycle arrest per se, but rather in profound changes in cellular metabolism, function, and secretory behavior, ultimately disrupting neuronal networks and homeostasis.

As the brain ages, senescent cells accumulate in key regions such as the hippocampus, prefrontal cortex, and basal ganglia, areas crucial for memory, executive function, and motor control [4]. These cells develop a pro-inflammatory and tissue-disruptive phenotype known as the SASP (Table 1). SASP is characterized by the secretion of inflammatory cytokines (e.g., IL-6, IL-1β, TNF-α), chemokines, matrix-degrading enzymes (MMPs), growth factors (e.g., VEGF), and reactive oxygen species (ROS) [43]. ROS and MMPs are also implicated in various neuroinflammatory disorders, whereas the origin and context of these processes distinguish age-related cognitive decline (ARCD). In contrast to disease-specific neuroinflammation, which arises secondary to distinct pathological triggers such as autoimmunity, protein aggregation, or acute injury, ARCD develops in the absence of overt neuropathology. It is primarily driven by chronic, low-grade, and systemic inflammation associated with aging (“inflammaging”). The gradual accumulation of senescent cells and their sustained secretion of SASP factors create a persistent yet subclinical neuroinflammatory environment. This long-term, diffuse exposure leads to oxidative stress, impaired synaptic plasticity, and reduced neurogenesis, ultimately contributing to cognitive decline. Thus, while the inflammatory mediators may overlap with those observed in other neuroinflammatory conditions, the chronicity, subtlety, and systemic nature of their activation are defining features of ARCD.

Moreover, SASP has a paracrine effect, spreading the senescent phenotype to neighboring cells through extracellular vesicles and signaling molecules [44]. This leads to a vicious cycle of tissue damage, amplifying senescence and functional decline. In neurodegenerative diseases such as Alzheimer’s disease (AD), Parkinson’s disease (PD), and frontotemporal dementia, the role of senescence is particularly pronounced [45].

Recent research has highlighted the critical role of senescence-associated secretory phenotype (SASP)-driven chronic inflammation in the progression of neurodegenerative diseases, particularly through the abnormal aggregation of key proteins such as tau, amyloid-beta, and alpha-synuclein [46,47]. These protein aggregates disrupt neuronal function and trigger reactive gliosis, leading to a self-sustaining cycle of neuroinflammation and neurodegeneration.

The results of the studies demonstrate that co-pathologies involving tau, amyloid-beta, and alpha-synuclein synergistically amplify neuroinflammatory responses, accelerating neuronal loss and cognitive decline. For example, co-expression of these proteins in animal models leads to robust activation of microglia and infiltration of tissue-resident memory T cells, which further exacerbate neuroinflammation and neurodegeneration [48,49]. This synergy is particularly evident in regions such as the hippocampus and cortex, where elevated protein pathology correlates with increased neuronal loss and immune activation.

Cellular senescence, especially in microglia and astrocytes, is increasingly recognized as a key driver of neuroinflammation in aging and neurodegenerative diseases. Senescent glial cells release SASP factors, including pro-inflammatory cytokines and chemokines, which perpetuate chronic inflammation and impair tissue repair mechanisms. Proteomic analyses reveal that aged brains exhibit upregulated SASP markers and increased expression of genes associated with microglial activation and senescence, further linking senescence to impaired remyelination and neurodegeneration [47,50].

Recent findings underscore the importance of microglia-astrocyte crosstalk in sustaining neuroinflammation [47]. Activated microglia and astrocytes release factors that amplify each other’s inflammatory responses, creating a feed-forward loop that drives chronic neuroinflammation and neurodegeneration. For instance, astrocyte-derived SFRP1 enhances microglial activation and cytokine secretion, contributing to the persistence and chronicization of neuroinflammatory processes [51].

The presence of senescent cells in the aging brain has been confirmed through molecular markers, including p16-INK4a, p21-CIP1/WAF1, and senescence-associated β-galactosidase (SA-β-Gal) activity [52] (Table 1). Elevated levels of these markers have been detected in both human post-mortem brain tissue and animal models of aging and neurodegeneration, particularly in brain regions critical for cognitive function [53,54]. Of note, p16-INK4a expression correlates with biological brain age and cognitive impairment severity, making it a candidate biomarker for senescence load in the CNS [53].

Senescent cells not only promote “inflammaging” but also impair brain plasticity by reducing the regenerative potential of neural stem cells (NSCs) and disrupting synaptic remodeling [55,56]. Recent studies show that aging and disease-related senescence in NSCs lead to metabolic abnormalities, disrupted protein homeostasis, and mitochondrial dysfunction, which collectively diminish neurogenesis and compromise cognitive functions such as learning and memory [57,58]. For example, research conducted by Faria et al. demonstrated that senolytic treatments in aged mice enhanced cognitive function by reducing inflammation and improving neuronal plasticity, highlighting the direct impact of NSC senescence on brain health [59].

Furthermore, senescence of endothelial cells weakens the blood–brain barrier (BBB), increasing its permeability and allowing harmful peripheral molecules and immune cells to infiltrate the central nervous system. This breakdown in vascular integrity is strongly linked to white matter lesions, cerebral small vessel disease, and vascular cognitive impairment [60]. It was found out that senescent endothelial cells in the cerebro-vasculature were associated with impaired neurovascular coupling, microvascular rarefaction, and BBB disruption, all of which contribute to cognitive decline [61]. Notably, senolytic interventions in animal models improved BBB integrity and cognitive performance, underscoring the therapeutic potential of targeting vascular senescence [61,62].

Given the multifaceted and causal role of cellular senescence in brain aging and neurodegeneration, these cells have emerged as attractive therapeutic targets. The development of senotherapeutic strategies aims to either selectively eliminate senescent cells (senolytics) or suppress their pro-inflammatory SASP (senomorphics) [63]. Preclinical studies have shown that such interventions can reduce neuroinflammation, restore synaptic function, and even reverse cognitive deficits in aged animal models [64,65]. By clearing senescent cells or modulating their secretory profile, it may be possible to enhance cognitive resilience, delay the onset of neurodegenerative diseases, and extend healthspan.

The therapeutic promise of senotherapeutics in the brain underscores the urgency of further characterizing the dynamics of senescence in the CNS, identifying cell-type specific biomarkers, and developing safe and effective interventions that can be translated into clinical settings. With continued research, targeting senescence could become a cornerstone strategy for managing brain aging and improving quality of life in aging populations (Table 1).

**Table 1:** A conceptual framework of cellular senescence in the aging brain.

Topic	Key Concept	Implications for Brain Aging	Current Challenges	Future Directions & Potential Solutions	Ref.
Classical view of senescence	Irreversible cell cycle arrest in proliferative cells (e.g., fibroblasts) due to damage, telomere attrition, or oncogene activation.	Protective short-term (tumor suppression); harmful long-term due to accumulation.	Oversimplified view; ignores senescence in non-dividing cells like neurons and glia.	Expand definition to include post-mitotic senescence, focusing on metabolic and secretory dysfunction.	[66]
Expanded view (CNS involvement)	Senescence occurs in non- and slowly dividing CNS cells (neurons, astrocytes, microglia, OPCs, endothelial cells).	Disrupts cellular metabolism, intercellular communication, and neural network function.	Difficult to distinguish from other age-related dysfunctions; lack of CNS-specific markers.	Develop and validate cell-type-specific senescence biomarkers and non-invasive imaging techniques (e.g., PET tracers for p16).	[67]
SASP	Secretion of pro-inflammatory factors (IL-6, IL-1β, TNF-α), proteases (MMPs), growth factors, and reactive oxygen species (ROS).	Drives chronic neuroinflammation, oxidative stress, and disrupts the neural environment.	The SASP is heterogeneous; some components may have context-dependent beneficial roles.	Develop next-generation senomorphics that selectively inhibit harmful SASP factors while preserving beneficial tissue-repair functions.	[68]
Paracrine Spreading	SASP factors and extracellular vesicles transmit the senescent phenotype to neighboring healthy cells (“bystander effect”).	Creates a feed-forward cycle, amplifying tissue damage and accelerating functional decline.	Hard to block systemic/paracrine signaling without causing off-target effects on healthy cells.	Develop localized delivery systems (nanoparticles) and agents that neutralize specific SASP factors or block their receptors.	[69]
Impact on Plasticity	Senescent cells reduce neural stem cell (NSC) regeneration and impair synaptic remodeling.	Loss of brain plasticity accelerates cognitive impairment and hinders recovery from injury.	Limited innate regenerative capacity in the adult human brain.	Combine senotherapeutics with pro-regenerative interventions (e.g., NSC transplantation, neurotrophic factors).	[56]
BBB Integrity	Senescent endothelial cells and pericytes increase blood–brain barrier permeability.	Allows infiltration of harmful peripheral molecules and immune cells, linked to vascular cognitive impairment.	Targeting vascular senescence without compromising systemic vascular health or causing toxicity.	Design endothelial-specific senolytics and utilize nanoparticle-based delivery to target the cerebrovasculature.	[70]
Therapeutic Implications	Senolytics clear senescent cells; Senomorphics suppress the SASP.	Preclinical evidence shows reduced neuroinflammation, improved proteostasis, and enhanced cognition.	Risk of off-target effects; incomplete clearance; long-term safety unknown.	Optimize therapy with refined senolytic cocktails, intermittent dosing regimens, and personalized medicine based on biomarker profiles.	[71]

## 3. Senotherapeutics: Classification and Mechanisms

Senotherapeutics represent a transformative and rapidly advancing field in biomedicine, offering novel therapeutic strategies to combat the cellular and molecular underpinnings of aging and age-related diseases. Central to this paradigm is the targeting of cellular senescence, a fundamental biological process in which cells irreversibly lose the ability to divide but remain metabolically active. While initially protective, preventing the proliferation of damaged or premalignant cells, senescent cells accumulate with age, contributing to tissue dysfunction, chronic inflammation, and the progression of various degenerative diseases, including those affecting the brain.

The goal of senotherapeutics is to restore tissue homeostasis and improve function by either eliminating senescent cells or neutralizing their harmful secretory profile, known as the SASP. Senotherapeutics are generally divided into two primary categories: senolytics, which induce selective death in senescent cells, and senomorphics, which modulate senescent cell activity without killing them. Each category has unique mechanisms of action, benefits, limitations, and therapeutic applications, especially within the delicate environment of the central nervous system (CNS).

### 3.1. Senolytics: Selective Clearance of Senescent Cells

Senolytic agents act by selectively triggering apoptosis in senescent cells (Table 2). It is worth noting that several additional compounds, including the dietary polyphenol fisetin, have shown senolytic activity under certain experimental conditions; however, their predominant effects are more accurately classified as senomorphic. Moreover, systemic regulators of metabolism and aging, such as insulin and components of the GH/IGF-1 axis, can indirectly shape the senescent cell landscape by modulating signaling pathways like PI3K/Akt. Despite these downstream influences, they are not considered true senolytic agents, as they do not directly induce the clearance of senescent cells.

Senescent cells evade death through upregulation of pro-survival signaling pathways, collectively termed senescent cell anti-apoptotic pathways (SCAPs) [71,72]. Key SCAPs include the BCL-2 protein family that governs mitochondrial apoptosis pathways; the PI3K/AKT/mTOR signaling axis, which promotes cell survival and growth; and the FOXO4-p53 interaction, which helps maintain senescent cell viability [73]. Senolytics disrupt these pathways, removing the pathological burden of senescent cells and alleviating the chronic inflammation and tissue remodeling they promote. Recent work continues to identify novel dependencies, such as the glutathione metabolism pathway, as critical for senescent cell survival [74].

**Table 2:** Selected senolytic agents with direct apoptotic mechanisms and their clinical applications.

Senolytic Agent	Primary Target/ Mechanism	Key Effects	Notes/Limitations	Clinical Implementations	Ref.
Dasatinib (D)	Tyrosine kinase inhibitor; disrupts SCAP signaling pathways	Clearance of senescent adipose, endothelial, and glial cells	Approved for leukemia; repurposed as senolytic; systemic off-target effects	Pilot clinical trials in idiopathic pulmonary fibrosis and other age-related conditions	[75]
Quercetin (Q)	Flavonoid; modulates PI3K/AKT and other pro-survival signaling	Anti-inflammatory, antioxidant, and senolytic activity	Natural dietary compound; limited bioavailability and BBB penetration	Used in combination with dasatinib in early-phase clinical studies	[14]
D + Q Combination	Synergistic targeting of multiple SCAPs	Reduces senescent glial cells, decreases neuroinflammation, improves cognition; decreases tau and amyloid-β burden	Synergistic effect stronger than single agents	Ongoing clinical trials in Alzheimer’s disease, frailty, kidney disease, and other age-related disorders	[76]
Navitoclax (ABT-263)	BCL-2 and BCL-XL inhibition	Potent induction of apoptosis in senescent cells	Dose-limiting thrombocytopenia due to platelet dependence on BCL-XL	Investigated in oncology; limited senolytic clinical use due to toxicity	[77]
FOXO4-DRI Peptide	Disrupts FOXO4–p53 interaction	Triggers apoptosis selectively in senescent cells; spares normal cells	Experimental; limited in vivo data so far	Preclinical stage; potential applications in neurodegeneration and cancer	[78]
Emerging strategies (nanocarriers, brain-penetrant prodrugs)	Targeted delivery platforms; enhanced BBB permeability	Improve selectivity and efficacy of senolytic agents	Early development; preclinical research phase	Not yet in clinical trials; potential to optimize CNS-targeted senolytic therapies	[79]

Among the most studied senolytics are dasatinib, a tyrosine kinase inhibitor originally used in cancer therapy, and quercetin, a naturally occurring flavonoid with anti-inflammatory and antioxidant effects [14]. When combined (D + Q therapy), these compounds exhibit synergistic senolytic activity, effectively clearing senescent cells from various tissues. In preclinical models of brain aging, D + Q therapy has been shown to reduce the load of senescent glial cells, dampen neuroinflammation, restore synaptic function, and enhance cognitive performance in tasks dependent on memory and learning [80,81]. Importantly, D + Q also reduces the accumulation of toxic protein aggregates, such as tau and amyloid-beta, commonly implicated in Alzheimer’s disease, thereby improving proteostasis and neuronal health [82,83].

Newer, second-generation senolytics aim to refine this therapeutic strategy by improving specificity, enhancing blood–brain barrier (BBB) permeability, and minimizing off-target effects. One such agent is navitoclax (ABT-263), which targets BCL-2 and BCL-XL proteins, though its clinical utility has been limited by thrombocytopenia due to platelet dependence on BCL-XL [84].

Another promising molecule is the FOXO4-DRI peptide [85], which selectively disrupts the FOXO4-p53 interaction, triggering apoptosis in senescent cells without affecting normal cells. The work by Kong et al., (2025) provides compelling evidence of FOXO4-DRI’s ability to induce apoptosis in senescent fibroblasts within highly recalcitrant keloid tissue. Their study identifies elevated p16 levels, increased SA-β-gal staining, and especially enhanced p53-Ser15 phosphorylation as defining features of the keloid senescent microenvironment. FOXO4-DRI treatment promoted the nuclear exclusion of p53-pS15, leading to a loss of the FOXO4-mediated survival signal that normally protects senescent fibroblasts from apoptosis. By reducing the proportion of G0/G1-arrested cells and triggering selective senescent-cell death, FOXO4-DRI effectively disrupted the chronic pro-inflammatory state that drives keloid persistence and recurrence. This study positions FOXO4-DRI as a targeted strategy for fibrotic dermal disorders where senescent cell accumulation contributes to pathological remodeling.

In a mechanistic complement to these findings, Bourgeois et al., (2025) [86] provide a detailed structural characterization of the interaction between p53 and FOXO4, along with the manner in which FOXO4-DRI disrupts this interaction. Using solution NMR, they demonstrate that the disordered p53 transactivation domain (p53-TAD2) binds to FOXO4 and that FOXO4-DRI can occupy this same interface, forming a transient but functionally meaningful complex. Importantly, they show that p53 phosphorylation increases the binding affinity of both FOXO4 and FOXO4-DRI, which parallels the elevated p53-pS15 observed in the keloid study by Kong et al. Their findings validate the central mechanism underpinning FOXO4-DRI’s senolytic activity: disruption of the FOXO4–p53 survival axis, leading to reactivation of apoptosis in senescent cells. By mapping this interaction at atomic resolution, Bourgeois and colleagues lay the groundwork for rational design of next-generation FOXO4-derived peptides and small-molecule senolytics aimed at diseases linked to chronic cellular senescence.

The study by Huang et al., (2021) extends the evaluation of FOXO4-DRI to the field of regenerative medicine, examining its effect on in vitro–expanded human chondrocytes, a cell population known to accumulate senescent cells during preparation for autologous chondrocyte implantation (ACI). Their findings confirm that FOXO4-DRI selectively eliminates senescent chondrocytes at high population-doubling levels (PDL9) while sparing minimally expanded cells (PDL3). Although FOXO4-DRI did not significantly enhance the intrinsic chondrogenic capacity of the surviving cells, the resultant cartilage tissue showed reduced expression of SASP factors, suggesting improved inflammatory and matrix-remodeling profiles. This study highlights both the potential and the limitations of FOXO4-DRI in regenerative applications: while the peptide effectively removes harmful senescent cells, additional interventions may be required to fully restore high-quality chondrogenesis in aged or extensively expanded cell populations.

Together, these three studies demonstrate a coherent mechanistic framework: FOXO4-DRI operates by uncoupling the FOXO4–p53 interaction that normally protects senescent cells from apoptosis.

Additionally, novel delivery systems such as nanocarriers and brain-penetrant prodrugs are under investigation to improve the targeted delivery of senolytics to senescent cells within the CNS [63].

It is important to distinguish the primary senolytic action of these drugs, the induction of apoptosis in senescent cells, from their subsequent anti-inflammatory effects. The reduction in neuroinflammation observed after senolytic treatment is primarily a downstream consequence of removing the source of the pro-inflammatory SASP, i.e., the senescent cells themselves. However, some senolytic agents, such as quercetin, also possess direct anti-inflammatory (senomorphic) properties that can contribute to the overall therapeutic benefit. The most definitive evidence for a true senolytic mechanism, as opposed to a purely anti-inflammatory one, is the direct demonstration of a reduced burden of senescent cells, quantified by a decrease in senescence markers (e.g., p16INK4a, SA-β-Gal) in target tissues following treatment. This confirmation is crucial for validating that the observed cognitive and functional improvements are indeed driven by the clearance of senescent cells.

### 3.2. Senomorphics: Modulating Senescent Cell Behaviour

Senomorphics, by contrast, do not eliminate senescent cells but instead modulate their harmful secretions, particularly components of SASP [71] (Table 3). This SASP suppression mitigates the inflammatory and tissue-damaging consequences of senescence while preserving beneficial cellular functions, such as trophic support and immune surveillance [33]. Senomorphics are especially relevant in the brain, where non-dividing neurons and glial cells can adopt senescent-like features, and indiscriminate removal of these cells could lead to disruption of critical neural circuits or loss of regenerative capacity [87].

Notable senomorphic agents include rapamycin [88], an mTOR inhibitor that suppresses SASP by downregulating pro-inflammatory cytokines (e.g., IL-6, IL-1β) and promoting autophagy, a process essential for cellular cleanup and homeostasis. Rapamycin has demonstrated neuroprotective effects in animal models by enhancing neuronal survival, reducing oxidative stress, and preserving cognitive function during aging [89]. Similarly, metformin, a widely prescribed antidiabetic drug, activates AMP-activated protein kinase (AMPK) and indirectly inhibits mTOR, yielding anti-inflammatory and neuroprotective effects [90]. Observational studies suggest that metformin use is associated with reduced cognitive decline and a lower incidence of neurodegenerative diseases [91].

**Table 3:** Summary of Selected Clinical Trials Involving Senotherapeutic Agents.

ClinicalTrials.gov Identifier/ Reference	Condition	Intervention	Phase	Primary Outcomes/Key Findings
NCT04785300/[92]	Mild Alzheimer’s Disease	Dasatinib + Quercetin (D + Q)	1	Feasibility and safety established; reduction in senescence and inflammation biomarkers in blood and CSF observed.
NCT04685590/[93]	Older Adults at Risk for AD	Dasatinib + Quercetin (D + Q)	Pilot	Improvement in cognitive and mobility measures compared to placebo.
NCT04313634 [94]	Diabetic Kidney Disease	Dasatinib + Quercetin (D + Q)	1	Ongoing; assessing safety and senescence biomarkers.
[95,96]	Healthy Elderly	Fisetin	2	Completed; results pending on safety and biomarkers of senescence and inflammation.
NCT04511416 [97]	Diabetes and Cognitive Decline	Metformin	N/A	Meta-analyses associate metformin use with a significantly reduced risk of cognitive impairment and dementia.
RTB-101-204 [98]	Aging	RTB101 (mTOR inhibitor)	2	Completed; reduced incidence of respiratory tract infections.

The study by Zimmerman et al., (JAMA Network Open, 2023) found that early cessation of metformin, without kidney dysfunction, was associated with a 21% increased risk of dementia compared to continued use [99]. Analyzing over 41,000 patients with diabetes, the authors showed that this association was not explained by changes in blood glucose (HbA1c) or insulin use, suggesting metformin’s potential neuroprotective effects beyond glycemic control. The findings highlight the risks of premature metformin discontinuation and support further research into its role in dementia prevention and healthy brain aging.

Another important class of senomorphics is JAK inhibitors, such as ruxolitinib and baricitinib, which block the JAK/STAT pathway, a key regulator of SASP gene expression [100,101]. In aged brain models, JAK inhibition attenuates microglial activation, decreases pro-inflammatory cytokine release, and may restore synaptic function and plasticity [102,103]. Additionally, natural compounds like fisetin and resveratrol have shown senomorphic potential by modulating oxidative stress responses and inhibiting NF-κB, a transcription factor involved in SASP regulation [104].

Senomorphics are particularly attractive for long-term administration, given their lower toxicity profiles and suitability for chronic conditions. They can be employed independently or in combination with senolytics to prime tissues for safer cell clearance, enhance therapeutic outcomes, and minimize inflammatory surges that may accompany massive senescent cell death.

Together, senolytics and senomorphics offer complementary strategies for brain aging intervention. While senolytics act as cellular rejuvenators, removing detrimental senescent cells, senomorphics serve as regulators, reducing the inflammatory and degenerative consequences of cellular aging. The choice of approach depends on multiple factors, including the type of senescent cell, disease context, stage of aging, and individual patient characteristics.

### 3.3. Preclinical and Clinical Evidence

A growing body of preclinical research has provided compelling support for the use of senotherapeutics as a promising strategy to counteract brain aging and neurodegenerative processes. In numerous animal models, especially aged rodents and transgenic models of Alzheimer’s disease (AD), interventions targeting senescent cells have yielded significant improvements in brain function and structure [105,106]. These studies not only validate the causal role of cellular senescence in cognitive decline but also demonstrate that pharmacological manipulation of senescent cells can be both feasible and beneficial.

In aged mice, treatment with the senolytic combination dasatinib and quercetin (D + Q) has been shown to significantly improve cognitive outcomes, particularly in tests of spatial learning, memory retention, and executive function [107]. Mechanistic investigations have revealed that these benefits are closely linked to the clearance of senescent glial cells, particularly astrocytes and microglia, which become dysfunctional with age and contribute to chronic neuroinflammation [108]. Following D + Q administration, researchers observed a reduction in senescence markers (e.g., p16-INK4a and SA-β-Gal activity) and a decrease in SASP-related cytokines, including IL-6, IL-1β, and TNF-α, within key brain regions such as the hippocampus and prefrontal cortex. This attenuation of neuroinflammation was accompanied by improvements in synaptic plasticity markers, such as increased brain-derived neurotrophic factor (BDNF) levels and enhanced long-term potentiation (LTP), further supporting the cognitive improvements noted.

Moreover, senolytic treatment has been shown to ameliorate pathological protein accumulation in the aging brain. For instance, in AD mouse models, D + Q reduced the burden of amyloid-beta plaques and hyperphosphorylated tau aggregates, potentially by restoring proteostasis mechanisms and reducing the inflammatory milieu that exacerbates protein misfolding [109]. These findings suggest that senolytics may not only improve function but also slow or reverse neurodegenerative pathology.

The study by Ruggiero et al. offers valuable translational insights into the effects of the senolytic combination dasatinib and quercetin (D + Q) in a nonhuman primate (NHP) model of aging [110]. As one of the first long-term senolytic trials in primates, it bridges the gap between rodent studies and human clinical research. In this six-month trial, middle-aged NHPs received monthly D + Q treatment, followed by a 10% caloric restriction (CR) in both treatment and control groups. The researchers evaluated markers of senescence, inflammation, immune function, and metabolic health. D + Q reduced senescence-associated gene expression in adipose tissue and lowered circulating levels of pro-inflammatory markers (PAI-1, MMP-9), indicating suppression of the senescence-associated secretory phenotype (SASP). Immune profiles revealed an anti-inflammatory shift and decreased markers of microbial translocation, indicating improved gut barrier function without significant changes to the microbiome. This study confirms D + Q’s potential to modulate key aging hallmarks and provides biomarkers for clinical monitoring. It also underscores the promise of combining senolytics with lifestyle interventions such as CR for enhanced outcomes.

Senomorphic agents have also demonstrated promising neuroprotective effects in preclinical models. Rapamycin, an mTOR inhibitor, is particularly well-studied for its ability to extend lifespan and healthspan across multiple species, including mice, rats, and non-human primates [111]. In the brain, rapamycin promotes autophagy, thereby enhancing the clearance of damaged organelles and protein aggregates, while simultaneously suppressing SASP expression and supporting neuronal viability. Studies have shown that long-term rapamycin treatment preserves cognitive performance in aged animals and delays the onset of neurodegenerative hallmarks, such as synaptic loss, tau phosphorylation, and gliosis [112].

Metformin, another senomorphic with AMPK-activating and indirect mTOR-inhibiting effects, has also been associated with cognitive preservation in aging rodents and lower incidence of dementia in human observational studies [113,114]. By reducing oxidative stress, enhancing mitochondrial function, and inhibiting pro-inflammatory pathways, metformin demonstrates a multi-faceted protective effect on the aging brain.

Building on these encouraging findings, the clinical translation of senotherapeutics has begun. Early phase clinical trials are now evaluating the safety, pharmacokinetics, and preliminary efficacy of senolytic agents such as D + Q and navitoclax in elderly individuals and patients with mild cognitive impairment (MCI) or early-stage Alzheimer’s disease [115,116]. These studies aim to assess tolerability, determine appropriate dosing regimens, and measure biomarker responses including changes in plasma neurofilament light chain (NfL), glial fibrillary acidic protein (GFAP), inflammatory cytokines, and cognitive test scores.

However, clinical application of senotherapeutics faces several important challenges. One major hurdle is ensuring effective delivery of therapeutic agents to the brain, given the restrictive nature of the blood–brain barrier (BBB). Many senolytics have limited CNS bioavailability, prompting the development of novel delivery systems, such as lipid-based nanoparticles, polymeric carriers, and prodrug formulations, designed to enhance brain penetration while minimizing systemic exposure and toxicity [63].

Additionally, concerns persist about long-term safety and off-target effects. While the elimination of harmful senescent cells can be beneficial, senescent cells may also have context-dependent physiological roles, such as in wound healing, tissue remodeling, and immune modulation. Indiscriminate clearance could impair these processes, particularly in tissues where senescent cells are functionally active, such as the neurovascular unit. Therefore, dosing strategies, including intermittent treatment schedules, tissue-targeted senolytics, and combination therapies with senomorphics, are being explored to maximize therapeutic benefit while mitigating risks [71,117].

Another significant obstacle is the lack of robust, validated biomarkers to monitor senescence dynamics in vivo. The development of fluid-based markers (e.g., circulating SASP proteins, exosomal miRNAs), molecular imaging tracers targeting senescence markers, and genomic profiling tools is essential to enable patient stratification, treatment monitoring, and personalized intervention protocols [118].

Despite these challenges, the field is progressing rapidly, fuelled by advances in aging biology, pharmacology, and biomarker discovery. The integration of senotherapeutics into clinical practice could represent a paradigm shift in aging medicine, offering a means to prevent or delay cognitive decline, improve quality of life, and reduce the burden of neurodegenerative diseases in the aging population. Continued interdisciplinary research, along with rigorous clinical trials, will be critical to realizing the full potential of senotherapeutics in brain aging management.

## 4. Challenges and Future Directions

**Figure 1:**
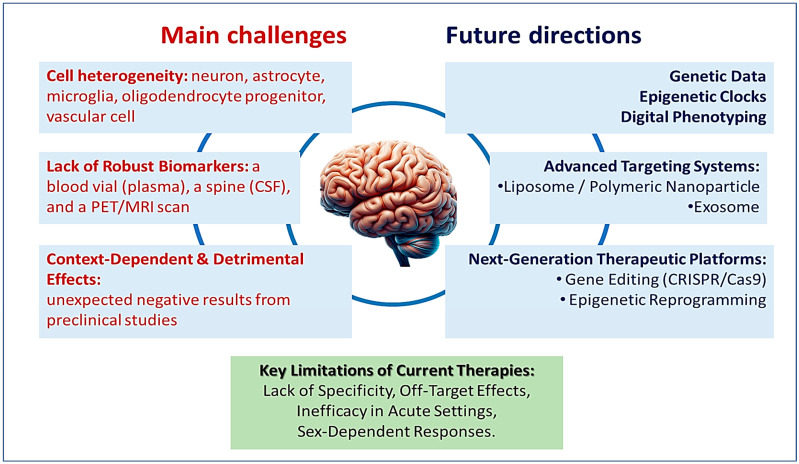
Challenges and future directions in the development of seno-therapeutic agents for the treatment of brain aging. The figure conceptualizes a necessary paradigm shift in central nervous system (CNS) therapeutics, moving from the current era of broad, ineffective strategies to a future defined by precision neurology. This transition is not incremental but foundational, demanding an integrated strategy that synergizes three critical pillars: deep genetic insights to understand disease at its root, sophisticated targeting systems to deliver therapies with cellular accuracy, and novel platform technologies to enact fundamental biological corrections.

Despite the growing body of evidence supporting the potential of senotherapeutics in managing brain aging, several significant scientific, clinical, and technological challenges remain to be addressed before these interventions can be widely implemented. A central obstacle is the heterogeneity of senescent cells, particularly within the complex cellular milieu of the central nervous system (CNS). Senescence manifests differently across neurons, astrocytes, microglia, oligodendrocyte progenitors, and vascular endothelial cells, with each cell type exhibiting distinct senescence-associated secretory phenotypes (SASP), metabolic profiles, and responses to stress [71,119]. This cellular diversity complicates therapeutic targeting, as a one-size-fits-all approach risks off-target effects or insufficient efficacy (Figure 1). A more refined understanding of cell-type specific senescence markers and context-dependent SASP signatures is needed to develop next-generation, precision senotherapeutics.

Another major challenge is the current lack of robust and non-invasive biomarkers for detecting and monitoring senescence in vivo, especially in the brain (Figure 1). Unlike peripheral tissues where biopsies and direct sampling are feasible, the brain presents unique limitations in accessibility. Advances in fluid-based biomarkers, such as cerebrospinal fluid (CSF) analysis, plasma assays for senescence-associated proteins, and circulating microRNAs, are under investigation, but none are yet fully validated for clinical use [38,65]. Additionally, neuroimaging modalities, including PET and MRI, are being explored for their potential to visualize senescent cell burden through molecular tracers targeting senescence markers like p16-INK4a or β-galactosidase activity [120].

Although the combination of dasatinib and quercetin (D + Q) has shown promise as a senolytic therapy in preclinical models, emerging research indicates that this approach may face significant limitations [121]. Recent investigations into senotherapeutics, particularly D + Q, have yielded nuanced and, at times, unexpectedly negative results, suggesting that the clearance of senescent cells is not universally beneficial across all tissues, conditions, and populations. Notably, some studies highlight critical challenges and potential risks associated with senolytic therapies in the context of aging and disease [81].

For instance, in work by Asha Rani and colleagues, the efficacy of D + Q was assessed in aging female rats to determine whether it could mitigate age-related cognitive decline [122]. Contrary to earlier findings in male rats, D + Q treatment failed to improve memory performance in females and did not prevent episodic memory deficits. The researchers proposed that hormonal changes associated with estropause, especially the decline in estradiol, may have influenced the response to treatment. These findings emphasize the need to consider sex-specific biological factors when evaluating anti-aging interventions and suggest that senolytic efficacy may not be uniform across sexes.

Similarly, a study by Blake Torrance and co-authors examined whether D + Q could rejuvenate immune function in aged mice exposed to influenza infection [123]. Despite theoretical support for the removal of senescent immune cells to enhance host defense, D + Q treatment produced no measurable improvements in viral clearance, T-cell responses, antibody production, or memory formation. This lack of effect challenges the assumption that senolytics can broadly enhance immune resilience in older individuals and suggests that the immune system may not benefit uniformly from senescent cell clearance during acute infections.

Another study by Antonio Battaglia-Vieni and colleagues investigated the use of D + Q in a mouse model of acute kidney injury (AKI) caused by folic acid toxicity [81]. Unexpectedly, D + Q not only failed to protect kidney function but also exacerbated tissue damage. The researchers observed increased markers of kidney injury, elevated levels of the senescence marker p21, and heightened pro-inflammatory activity. In addition, expression of the anti-aging protein Klotho was reduced. These results suggest that in the context of acute injury, senolytics may disrupt essential repair processes or induce off-target damage, underscoring the importance of therapeutic context and timing in the use of senotherapeutics (Figure 1).

Taken together, these studies underscore that senolytic therapies are highly context-dependent, with their effects influenced by factors such as sex, organ-specific biology, and disease state. While senolytics may offer therapeutic benefits in managing chronic, low-grade aging processes, their application in acute injury settings or hormonally dynamic systems may be ineffective or even harmful. These findings highlight the urgent need for more selective biomarkers to guide senolytic interventions, the development of next-generation senolytics with improved specificity and safety profiles, and enhanced delivery methods capable of targeting specific cell populations.

In this context, personalized medicine approaches may play a pivotal role in enhancing the precision and safety of senotherapeutics. By integrating genetic information (e.g., APOE genotype), epigenetic clocks (e.g., DNA methylation-based estimations of biological brain age), and digital cognitive phenotyping (e.g., wearable-derived data on gait, speech, and sleep), clinicians may be able to stratify patients into those most likely to benefit from specific senolytic or senomorphic therapies [124,125]. Such stratification would enable tailored treatment regimens, optimizing dosage, timing, and therapeutic combinations based on individual aging trajectories.

Emerging technologies and delivery systems hold promise for overcoming key limitations in the field. For instance, nanoparticle-mediated drug delivery, including liposomes, polymeric nanoparticles, and exosome-based platforms, are being designed to cross the blood–brain barrier efficiently and deliver senotherapeutic agents directly to target sites within the CNS [126]. These approaches may enhance bioavailability, reduce systemic toxicity, and allow for controlled release of therapeutics. Additionally, gene editing tools such as CRISPR/Cas9 offer opportunities to selectively silence or repair genes associated with senescence pathways, while epigenetic reprogramming strategies, including transient expression of Yamanaka factors or histone-modifying enzyme modulators, are being explored to reverse cellular aging without inducing tumorigenesis or loss of cell identity [127,128].

A promising future direction lies in the development of combination therapies, in which senolytics or senomorphics are integrated with lifestyle interventions known to modulate the biology of aging. Evidence suggests that physical exercise, caloric restriction, cognitive training, and sleep optimization can have a positive impact on key processes, including neuroinflammation, mitochondrial function, and synaptic plasticity [129,130]. When combined with pharmacological interventions, these approaches may exert synergistic effects, fostering greater brain resilience and supporting healthy cognitive aging more effectively than monotherapy alone.

Despite the inherent complexities of clinical translation, the landscape of senotherapeutics in brain aging remains highly promising. Progress in this field will depend on multidisciplinary collaboration across neuroscience, pharmacology, bioengineering, and data science, enabling refinement of therapeutic strategies, innovation in delivery technologies, and the establishment of reliable biomarkers. Such efforts hold the potential to deliver safe, effective, and personalized interventions that directly address one of the most fundamental drivers of cognitive decline in the aging brain.

## 5. Conclusions

Senotherapeutics offer a promising and innovative approach to managing brain aging and its associated cognitive decline. By targeting the fundamental process of cellular senescence, either through selective elimination of senescent cells or modulation of their harmful secretions, these interventions have demonstrated the ability to reduce neuroinflammation, improve synaptic function, and enhance cognitive performance in preclinical models. Although clinical translation is still in early stages, ongoing trials and emerging delivery technologies provide a pathway toward safe and effective use in humans.

Challenges such as blood–brain barrier permeability, senescence biomarker development, and long-term safety must be addressed through continued research and technological advancement. Personalized approaches that integrate genomics, epigenetics, and digital health data may further refine the application of senotherapeutics, ensuring maximal benefit with minimal risk. As our understanding of brain aging deepens, senotherapeutics may become a central pillar of precision medicine aimed at prolonging cognitive healthspan and improving quality of life in aging populations.

## Data Availability

No new data were created or analyzed in this study. Data sharing is not applicable to this article.
